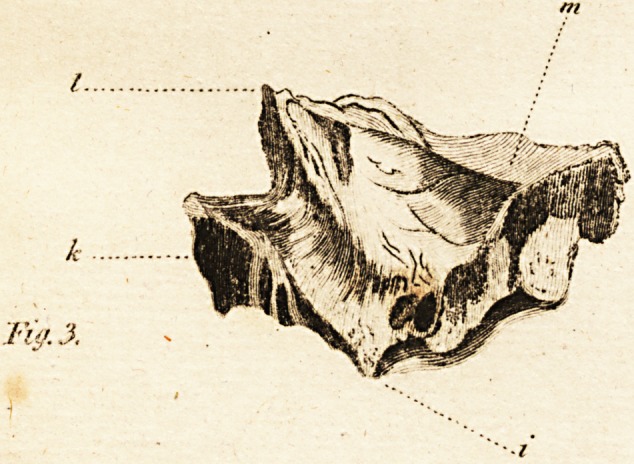# To the Editors of the Medical and Physical Journal

**Published:** 1801-06

**Authors:** John Bailey

**Affiliations:** Harwich


### Medical and Physical Journal.

VOL. V.] JUNE, 1801. [NO. XXVIII.

### *To the Editors of the Medical and Physical Journal.*

GENTLEMEN,

ENCLOSED is a drawing of a very curious and extensive Exfoliation, which I send you for publication, if you think it of note sufficient to occupy a place in your Journal.

I am, &c.

*Harwich, April,* 1801. JOHN BAILEY.

---

As no practical inferences can be deduced from this case, its curiosity alone attracting observation, I shall, as briefly as possible, narrate its history.

Thomas Lay, the subject of this case, is a child of three and a half years of age; in September last he was attacked with measles, which were accompanied by a soreness of the throat, as is not unfrequently the case, and a slight exulceration of the gums on the right side. From the inattention and uncleanliness of the nurse, the matter which exuded being suffered to remain, entirely eroded the gums; the teeth, losing their support, fell from the aveolar cavities, which were now left perfectly bare. At this period, which was more than two months subsequent to the attack of measles, I was requested to give my assistance.—On touching the parts with my finger, I found they admitted of much motion. The skin of the cheek was reddened and tumefied, and an offensive discharge issued from the mouth and right nostril. Some simple detergent application was used; decoctum cinchonæ cum acido sulphurico was exhibited internally. After pursuing this plan for a week or ten days, the bone protruded so far as to impede the shutting of the mouth. From this time I began to loosen it daily with a pair of pocket forceps, and at the lapse of a fortnight withdrew a complete half of the maxilla superior, of which the drawing is a faithful representation: a slight hæmorrhage followed, the aperture in the mouth soon closed, and the tumefaction of the cheek subsided.

The loss of so considerable a portion of bone, it might be expected, would occasion a good deal of deformity; but this is by no no means the cafe: there remains no further external veftige of the difeafe than a flight retraction of the angle of the mouth.

REFERENCES TO THE PLATE.

FIG. I. fhews the bone in fitu.
*a.* The os nafi
*b* The fymphyfis
*c.* The alveolar cavities
*d.* The infra orbitar foramen
*e.* The orbitar procefs.

FIG. II. gives an under view of the bone.
*f.* The fymphyfis
*gg.* The os palati
*h.* The alveolar cavities

FIG. III. gives an internal view.
*i.* The os palati
*k.* The fymphyfis
*l.* The nafal procefs
*m.* The orbitar procefs, which when the bone was recently taken away, was nearly entire, but was broken off by an accident afterwards.

## Figures and Tables

**Fig. 1. The Bones in Situ f1:**
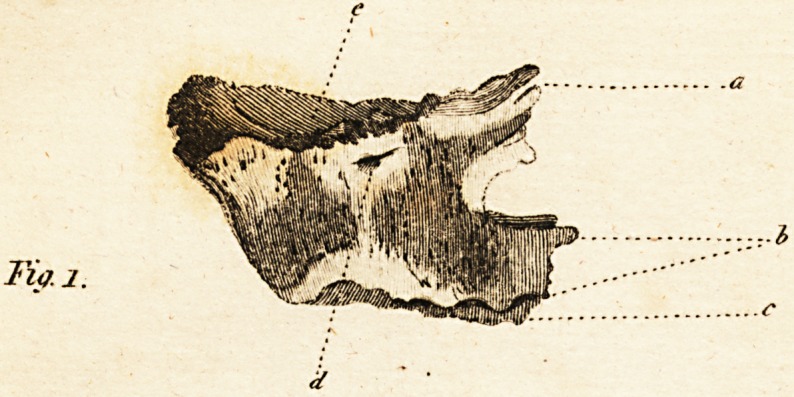


**Fig. 2. An Under View of the Bone f2:**
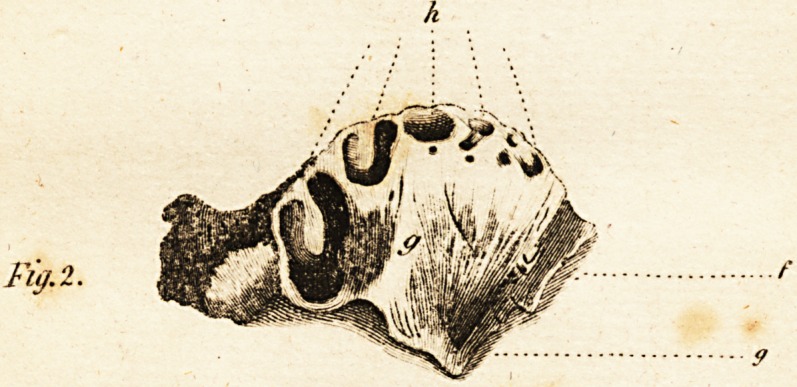


**Fig. 3. An Internal View f3:**